# Mental health in criminal justice: presentations, provision and possibilities

**DOI:** 10.1192/bjb.2024.79

**Published:** 2024-10

**Authors:** Andrew Forrester

**Affiliations:** Division of Psychological Medicine and Clinical Neursciences, School of Medicine, Cardiff University, UK

**Keywords:** Criminal justice, prison, courts, police, complexity

## Abstract

This article summarises the *BJPsych Bulletin* 2024 special edition on mental health in criminal justice and correctional settings. The edition considers issues across a range of settings, including police custody, the courts and prisons, as well as considering wider international questions and systems within the field. In this edition, we assert the right of the individual to healthcare services that should be available, accessible, acceptable and of good quality. Psychiatry must play a significant role in shaping this debate as it moves forward.

Welcome to this special edition in which we focus on mental health in criminal justice and correctional systems, with a focus on how people present clinically, the range of services that are and could be provided and limitations and possibilities within the field.

It is immediately apparent that this area is complex. From a clinical perspective, presentations are often multiple and serious, with over-representation of illness across domains of physical health, mental health and substance misuse.^1^ From the perspective of service provision, healthcare services must operate alongside and within other systems – such as prisons, courts and police custody – that they do not directly manage or run but nonetheless also provide a vital public service. How best to do this has historically been the subject of considerable discussion, yet at a time when the global prison population continues to rise, and seems set to rise further, we must continue to seek solutions. The current global prison population is likely in excess of 11 million people, albeit with considerable inter-regional and international variation.^2^

## The special edition

In this edition, Andrew Carroll and Adam Brett deal with this issue head-on, argue that ‘Jailing is failing’^3^ and describe a key role for psychiatry in a revised system through tiered models of prevention. While it is the case that we as psychiatrists have much to offer in the onward management of people with offending behaviour and mental illness, there is an abiding sense that we are not as fully engaged, or used as effectively, as we might be. How to solve this issue is likely as much about testing new service models working across health and justice areas as it is about our willingness to engage and become part of the solution.

Lara Arsuffi et al^4^ go on to describe one such model – Mental Health Treatment Requirements (MHTRs) – which we know can be successful when applied to people who have primary care-level mental health needs. Yet despite this, there is much to learn, and although MHTRs offer an alternative to imprisonment for appropriate cases – providing a framework through which onward care and supervision can be applied – there are some abiding questions. How and when should we apply them to people with severe and enduring mental illness, what barriers might prevent their proper use and how might such recommendations best sit within existing liaison and diversion services?^5,6^ Here, there is a clear role for future research.

A further model is described by Manuela Jarrett et al,^7^ who have evaluated the application of screening and brief interventions for alcohol-related conditions in police custody. While the use of health screening in police custody has significant support within the literature,^8^ the authors found that considerable challenges apply when attempting to implement a public health intervention model in these settings, and that any further changes are likely to require a significant training investment.

In further work aimed at improvements on a public health scale, Rebecca Crook at al^9^ have described a logic model with implementation strategies, as part of their project that aims to reduce and prevent suicide in prisons. Here, again, the complexity of clinical work in prisons is apparent, with a range of outer or environmental considerations (e.g. environmental instability, or the prioritisation of security concerns over the delivery of healthcare services) and internal, or individual, factors (e.g. previous experience of healthcare, or trust in institutional and health delivery systems). This model has a real potential strength, in that it tries to capture the complexity that is apparent in real-life systems and interactions, and promising further developments are suggested. In any case, there is no doubt that improvements are required in this area, given the most recent statistics from England and Wales, showing 371 deaths in custody in 1 year, of which 86 were self-inflicted.^10^

In a further demonstration of the complexity that characterises this broad field, Bradley Hiller et al^11^ identify issues that arise with chemsex, with evidence of a subgroup who engage in offending behaviour and also experience harm. To date, although there has been increasing recognition of the difficulties arising in this area, there has been no over-arching multi-agency strategy. So, Hillier et al have set about trying to change this by taking a three-pronged approach to key recommendations. These recommendations – training, research and network development – have broad support, and now require a framework to move forward.

There are also two further international contributions to this edition. The first – from Roland Jones and Alexander Simpson^12^ – considers issues arising with Medical Assistance in Dying (MAiD), when this is used amongst people in prison. While this area is ethically complex, the main arguments in support of access have been based upon the principle of equivalent care – that is, that prisoners should have the same standard of healthcare that is available in the wider community.^13^ The authors go on to describe four powerful cases, and argue that although this is an evolving area, considerable caution is required. Their clear recommendations deserve further thought.

The second – an interview of Professor Guillermo Rivera, who is Professor of Abnormal Psychology in Santa Cruz, Bolivia, by Claire McKenna^14^ – provides a fascinating glimpse of prison conditions and prison mental health services, in Bolivia. However, despite well documented and often dangerous prisons, and *de facto* self-governing prison systems, Rivera draws our attention to significant humanitarian efforts being made on the ground to improve and reform existing conditions. From a psychiatric perspective, only 1 of the 90 prisons in Bolivia has a psychiatrist who is funded by the State, and most prisons have no mental health staff at all. Yet, Rivera faces these issues head-on and takes an admirably practical approach to continuous improvement. His work is important.

In working to improve services within criminal justice and correctional settings, we set out clear progressive intent, and in undertaking this work we assert the right of the individual to healthcare services that should be available, accessible, acceptable and of good quality, irrespective of their legal status.^15^ Where, how and when this can best be delivered is likely to remain the subject of societal and political debate, yet medicine – and psychiatry in particular – must play a significant role in shaping this debate as it moves forward.

## Data Availability

Data availability is not applicable to this article as no new data were created or analysed in its preparation.

## About the author

**Andrew Forrester** is Professor of Forensic Psychiatry in the Division of Psychological Medicine and Clinical Neurosciences, School of Medicine, Cardiff University, UK, and a consultant forensic psychiatrist with Swansea Bay University Health Board, Swansea, UK and Oxleas NHS Foundation Trust, Dartford, UK, where he is also Director of Research.
